# Debris-covered glacier identification in the Karakoram aided by thermal remote sensing

**DOI:** 10.1016/j.isci.2026.115148

**Published:** 2026-03-13

**Authors:** Drolma Lhakpa, Mengmeng Li, Basang Tsedan, Tsomo Yongqing, Bin Cheng

**Affiliations:** 1 Tibet Institute of Plateau Atmospheric and Environmental Sciences, Tibet Meteorological Bureau, Lhasa 850000, China; 2 Key Laboratory of Atmospheric Environment of Tibet Autonomous Region, Lhasa, China; 3 School of Mathematics and Statistics, Zhengzhou University, Zhengzhou, China; 4 Henan Academy of Big Data, Zhengzhou University, Zhengzhou, China; 5 Meteorological Observatory, Tibet Meteorological Bureau, Lhasa 850000, China; 6 Lhasa Qinghai-Tibet Plateau Research Center, Lhasa 850000, China; 7 Finnish Meteorological Institute, Helsinki, Finland

**Keywords:** earth-surface processes, glacial landscapes, remote sensing

## Abstract

Glacier changes in the Karakoram Mountain (KM) region were analyzed using Landsat images from 2000 to 2021. Clean ice boundaries were mapped using supervised classification, while debris-covered ice was identified by integrating surface temperature, temperature vegetation dryness index (TVDI), and slope (ASTER DEM data). Extraction accuracy was 98% for clean ice and 67%–71% for debris-covered ice. In 2021, debris-covered ice represented about 5% of the total glacier area. Glacier area declined from 26.59 × 10^3^ km^2^ (2000) to 20.66 × 10^3^ km^2^ (2021), except for Amu Darya Basin (+1%). Tarim Basin and Indus Basin retreated by 14% and 25%, respectively. Rapid glacier loss in the Indus Basin may sharply decrease future river flow, underlining the need for accurate debris-covered glacier mapping for water resource assessment.

## Highlights

• Quantified the identification rate of glacier debris for each parameter
• Debris-covered glaciers make up about 5% of the total glacier area in the Karakoram region
• The average glacier area showed a decreasing trend in the Karakoram during 2000–2021
• Glacier retreat in the Indus Basin will greatly reduce future water resource availability

## Graphical abstract

**Figure:**
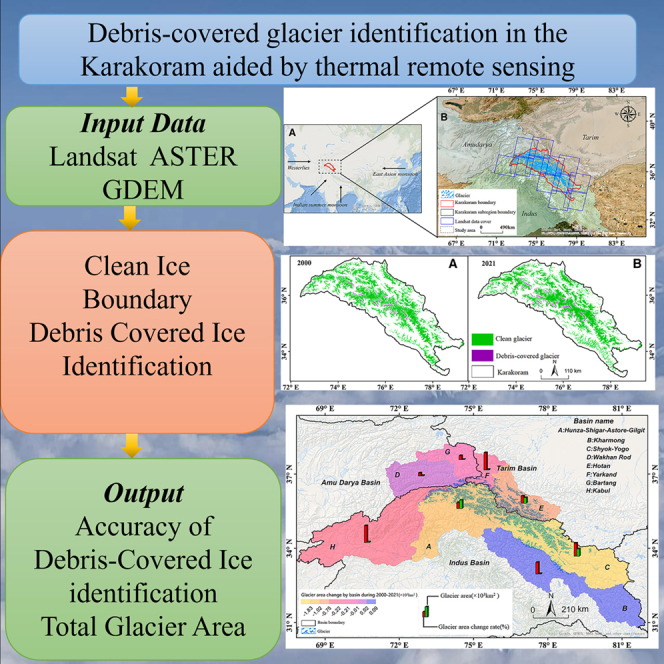

## Introduction

Glacial melting is accelerating, and ice mass loss exhibits significant regional heterogeneity at the global scale.^1^^,^^2^^,^^3^ The high-mountain Asia (HMA) region contains the largest ice volume outside the polar regions and plays a crucial role in shaping the hydrosphere and cryosphere. The interaction between glaciers and the atmosphere has intensified due to air temperature increase, which occurs at twice the global average in the HMA region.^4^^,^^5^^,^^6^^,^^7^ Furthermore, glacial meltwater provides essential water resources for ensuring food security, regional peace, and potential natural hazard protection for local residents and countries.^8^^,^^9^^,^^10^ Notably, the variations in glaciers across different subregions of HMA are considerable, with significant anomalies. A better understanding of the glacier mass and extent is crucial for predicting future glacier dynamics and evaluating broader environmental impacts.^11^^,^^12^

Recent research on glaciers in the Karakoram Mountain (KM) region has mostly focused on glacial mass changes, whereas systematic investigations of area variations since the 21st century remain limited.^13^^,^^14^ To address the complex spectra of glaciers and topographic characteristics of the region, semiautomated methods have largely been employed for glacier zone delineation.^15^^,^^16^^,^^17^ Glacier surfaces are typically categorized into three zones: snow/firn, clean ice, and debris-covered ice. Boundary identification for snow/firn and clean ice zones relies primarily on reflectance properties,^18^^,^^19^ with evolving techniques transitioning from traditional band ratio methods (Landsat Thematic Mapper [TM]/[ETM+]) and multitemporal minimum normalized difference snow index (NDSI) compositing techniques to innovative approaches that entail the integration of high-resolution imagery and deep learning algorithms, thereby significantly increasing the boundary extraction accuracy in small-scale studies.^16^^,^^17^^,^^18^^,^^19^^,^^20^^,^^21^^,^^22^

Notably, debris-covered ice, accounting for approximately 10% of the total glacier area, poses mapping challenges.^23^^,^^24^ Spectral limitations due to similarities between debris and rock/soil surfaces have driven researchers toward multitechnique integration.^25^ Approaches that combine radar imagery (advanced land observing satellite [ALOS]-1 phased array-type L-band synthetic aperture radar [PALSAR]-1) with manual delineation enable the establishment of precise glacier inventories^26^^,^^27^ but are limited by the spatial-temporal coverage and labor intensity. Emerging methods incorporating topographic parameters (e.g., slope), thermal infrared data (advanced spaceborne thermal emission and reflection radiometer [ASTER] temperature reduction indices), and multisource features (reflectance, humidity, or terrain) demonstrate enhanced potential for debris-ice identification. A growing consensus highlights an integrated analysis of multidimensional surface characteristics as a critical pathway toward achieving precise glacier mapping.^28^^,^^29^^,^^30^

The KM has been a focal point in glaciological research, particularly following the identification of the KM anomaly phenomenon. In this study, maximum likelihood classification (MLC), combined with manual delineation, was employed to accurately define clean ice boundaries. The impact of the quality of the original remote sensing images on the identification of clean glaciers was systematically evaluated. Furthermore, the surface temperature, temperature vegetation dryness index (TVDI), and slope were incorporated to effectively map debris-covered ice. The accuracy and feasibility of this approach were critically assessed by comparing the results with published glacier inventories. For surface temperature extraction, particular attention was given to the thermal characteristics of clean ice, debris-covered ice, a mixture of clean and debris-covered ice, and nonglacial surfaces. The use of this methodology significantly enhances the accuracy of temperature retrieval for debris-covered glaciers. Furthermore, to precisely extract the TVDI associated with supraglacial debris, representative samples were selected on the basis of the distinct moisture properties of glacier surface debris, which exhibits higher humidity than the adjacent terrain. Specifically, mixed-type debris glaciers on sun-facing slopes and fully debris-covered glaciers on shaded slopes were analyzed to systematically characterize the TVDI signatures of supraglacial debris.

The mountain topography of the Tibetan Plateau is a main condition, and it controls snow cover on the surface, thereby facilitating the development of glaciers.^31^ A quantitative assessment was conducted to evaluate the challenges posed by three critical factors—land surface temperature (LST), TVDI, and slope gradient—in extracting glacier surface debris. This analysis provides essential insights into increasing the debris identification accuracy, thereby establishing a methodological foundation for more precise supraglacial debris extraction in future studies. These findings advance the development of robust techniques for glacier debris mapping and contribute to an enhanced understanding of the glacier mass balance dynamics. Notably, glacial meltwater from the KMs serves as a vital water source for the Indus, Amu Darya, and Tarim River basins. Glacier evolution has significant implications for water resource utilization and disaster prevention. Owing to the gradual changes in regional glaciers, multiyear interval monitoring can reveal their transformation patterns. Via the use of Landsat data, clean and debris-covered glaciers were distinguished and integrated, and the accuracy of the results was assessed. The area of Karakoram glaciers in 2000 and 2021 was analyzed, with the above two temporal datasets effectively reflecting the overall trend in glacial changes over the 21-year period, demonstrating high temporal representativeness. Satellite images from September to December with minimal cloud cover and snowfall impacts were selected as reference data for determining annual glacial changes to reduce errors resulting from employing the original remote sensing data. Methodologically, widely recognized glacier identification techniques were employed, thereby ensuring both the validity of the glacial area change assessment methods and the reliability of the research data. This approach provides a robust technical guarantee for the accuracy of the findings.

Under global warming, glaciers are generally exhibiting a retreat trend. However, the KM region displays an anomalous phenomenon of “relatively stable or even locally expanding glaciers” (the “Karakoram Anomaly”). Since its revelation by satellite observations at the turn of the 21st century, this phenomenon has made the KM a hot research area for global glacier studies. Detailed, long-term monitoring and research of Karakoram glaciers are not only an urgent necessity for regional sustainable development but also a core element in the scientific exploration of global climate change frontiers. The river basin of Karakoram, especially the Indus River Basin with more than 300 million people in India and Pakistan, is facing severe challenges to its water sharing arrangements due to the accelerating impacts of climate change and extreme debris cover variations. Their flow is heavily dependent on snow and glacial melt, and climate change is causing an initial increase in river flows due to rapid glacial melt, but this is projected to be followed by a long-term, drastic reduction in water availability as the glaciers deplete or loss as observed. Hence, through this study, we highlight changes in the glacier area across various basins of the Karakoram over the past 21 years , providing essential reference data for protecting water sources in the headwater areas of these basins. The findings revealed that, compared with the glacial changes in other regions of HMA, Karakoram glaciers exhibited relatively minor area changes, with lower ablation rates. Additionally, in the identification of glacial debris cover, the debris moisture content on sun-facing slopes was lower than that on shaded slopes. This distinction could serve as a critical criterion for differentiating between fully debris-covered glaciers and mixed debris-covered glaciers. The primary objective of this study was to better understand the Karakoram anomaly phenomenon. The research findings can offer critical scientific evidence for downstream countries to formulate long-term water resource management strategies and prepare for potential water crises.

## Results

### Study area

**Figure 1:**
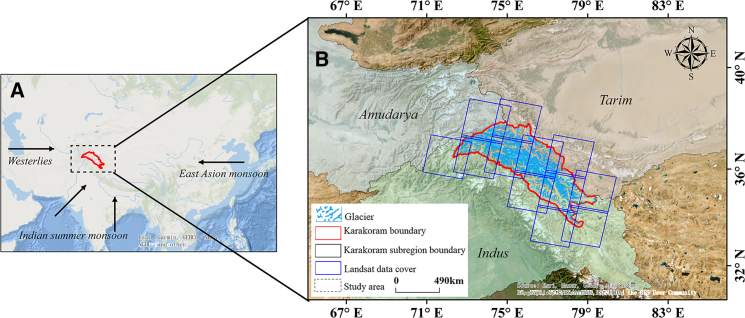
Geographical location of the study area (A) Geographical location of the KM (dashed frame) within the Asian continent. The area enclosed by the red boundary represents the study domain. The arrows indicate the prevailing wind direction toward the KM. (B) A zoomed-in map of the KM. The black boundary delineates the three watershed basins (Amu Darya, Tarim, and Indus) within the KM. Blue rectangles indicate the coverage of Landsat tiles. The spatial resolution of the Landsat data is 30 m).

The KM, which is situated in the northwestern part of the Tibetan Plateau and spans Pakistan, India, and China, with neighboring regions of Afghanistan and Tajikistan (Figure 1), receives a significant portion (70%–90%) of the total annual precipitation from Indian summer monsoon rainfall. Regional precipitation serves as the primary material supply source for glacial accumulation zones, whereas meltwater runoff constitutes the critical hydrological supply source for downstream river networks. At the basin scale, the glacier distribution exhibits notable spatial heterogeneity: the Indus River Basin, with the highest glacial coverage density (83%), functions as the principal solid reservoir in the region, and its meltwater runoff sustains transboundary rivers crucial to Pakistan and India; the glaciers in the Tarim River Basin, serving as the core water source for the largest inland basin of China (the Tarim Basin), maintain the hydrological equilibrium of oasis ecosystems; although the Amu River Basin exhibits the lowest glacier area proportion (3%), its meltwater provides the main baseflow contribution to the mainstream of the Central Asian Amu River.^32^

### Debris-covered glacier distribution

In 2021, debris-covered ice areas accounted for approximately 5% of the total glacier area in Karakoram. With respect to the debris-covered ice area coverage in the 3 basins of Karakoram, the results demonstrated that the Indus Basin exhibited a larger debris-covered ice area than the that in the Tarim Basin and Amu Darya Basin.

From 2000 to 2021, the debris-covered ice areas in different elevation bands increased, and the largest increase was obtained in the elevation range of 2,500–3,000 m (432.01 ± 37.44 km^2^), followed by 4,000–4,500 m (170.35 ± 13.62 km^2^) and 4,500–5,000 m (128.22 ± 7.34 km^2^). The elevation bands above 5,500 m and below 2,500 m yielded the smallest increases (<1 km^2^), and the remaining elevation bands generally exhibited increases ranging from 19 to 60 km^2^. With respect to the debris-covered ice area variation on different slopes, the results revealed that debris-covered ice area increase occurred mostly between slopes of 0° and 10° (10–66 km^2^), and the remaining slope range exhibited a debris-covered ice area increase less than 5 km^2^. With respect to the debris-covered ice area changes on the northern and southern slopes, the southern slope exhibited a larger debris-covered ice area, whereas with respect to the increase in the debris-covered ice area, that on the northern slope (51.18 ± 4.01 km^2^) was greater than that on the southern slope (48.48 ± 5.19 km^2^).

### Glacier change

**Figure 2:**
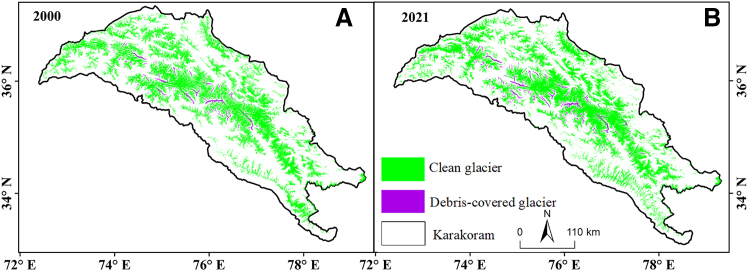
Clean and debris-covered glaciers in Karakoram after classification (A) Classification results for 2000. (B) Classification results for 2021.

According to the final classification results (Figure 2), the identification of glaciers in the KM Range was relatively ideal. Debris cover was mainly distributed at the glacier terminus and in ice tongue areas, as shown in the purple portion of the figure, and was primarily concentrated in the western to central regions of the KM Range. There were no significant changes in the distribution of clean glaciers between 2000 and 2021.

According to the RGI, Regions 14–02 pertain to the Karakoram glaciers, which are located in the western part of South Asia, with a recorded glacier area of 21,675 km^2^. The findings of this study revealed that the glacier area in the KM region was 20,660 km^2^ in 2021. The changes in the glacier area are reasonable compared with those in the RGI. Furthermore, research has shown that since 1970, the average glacier area change in Karakoram has not been significant. The region is primarily distinguished by a minimal rate of glacier area change, estimated at approximately 10%. Some glaciers demonstrate a reduction in the area change rate, and instances of glacier advance have partially mitigated the overall decrease in the glacier area. Most studies have indicated that the area change rate of Karakoram glaciers is also decreasing.^33^^,^^34^ The results of this study revealed that the area change rate of glaciers in concentration area is 11%, which is like previous findings and further indicates that the glacier area in the Karakoram approaches stability.

The Indus Basin is the largest glacierized region relative to the Tarim and Amu Darya Basins. The glacier area in the Tarim Basin and Indus Basin decreased, whereas that in the Amu Darya Basin increased. Moreover, the highest glacier area change ratio and annual mean glacier area change ratio values occurred in the Indus Basin. The Amu Darya Basin exhibited the lowest degree of change in the glacier area (Table 1). The specific performance in the Amu Darya Basin, Tarim Basin, and Indian Basin were 0.65 × 10^3^ ± 0.02 × 10^3^ km^2^, 6.77 × 10^3^ ± 0.2 × 10^3^ km^2^, and 19.17 × 10^3^ ± 0.57 × 10^3^ km^2^, respectively, in 2000 and 0.66 × 10^3^ ± 0.02 × 10^3^ km^2^, 5.79 × 10^3^ ± 0.17 × 10^3^ km^2^, and 14.21 × 10^3^ ± 0.43 × 10^3^ km^2^, respectively, in 2021. From 2000 to 2021, the glacier area in the Amu Darya River Basin increased slightly, and the corresponding glacier area change ratio and annual average glacier area change ratio were 1% and 0.07%/yr, respectively. The glacier area changes in the Tarim River Basin and Indus River Basin decreased by 0.97×10^3^ ± 0.27 × 10^3^ km^2^ and 4.96 ± 0.72 × 10^3^ km^2^, respectively. The corresponding glacier area change ratios were 14% and 25%, respectively.

**Table 1:** Glacier area and its changes in the Amu Darya, Tarim, and Indus basins

Drainage basin	Area (×10^3^ km^2^)		Area change (×10^3^ km^2^)	Area change rate (%)	Area change rate of year (%/yr)
	2000	2021			
Amu Darya	0.65 ± 0.02	0.66 ± 0.02	+0.009 ± 0.03	1	0.07
Tarim	6.77 ± 0.2	5.79 ± 0.17	−0.97 ± 0.27	14	0.69
India	19.17 ± 0.57	14.21 ± 0.43	−4.96 ± 0.72	25	1

The glacier area and its uncertainty are expressed in units of 10^3^ km^2^; “+” indicates an increase, and “−” indicates a decrease.

The glacier area results obtained are consistent with the glacier mass balance trends in the three basins calculated by Burn et al. (2017).^4^ over the same period, and they concluded that the glacier quality in the Indus River Basin decreased each year from 2000 to 2016 (4.0 ± 2.0 Gt).

The Indus River Basin boasts the most extensive glacier coverage in the KMs and is also one of the world’s most concentrated regions of glacier moraine. Iconic Karakoram glaciers such as the renowned Baltoro Glacier originate in this basin. Research indicates that the thickness of glacier moraine in the Indus River Basin exhibits pronounced spatial variability. In the high-elevation upstream areas, the moraine layer is generally thin, often less than 0.1 m in thickness. However, as glaciers flow downstream, under the combined influences of the “conveyor belt effect” and decreasing glacier velocity, moraine material gradually accumulates, forming a thick cover—sometimes several meters deep—at the glacier termini.^30^^,^^34^^,^^35^ This thick moraine layer provides effective thermal insulation for the glacier termini, significantly slowing their rates of ablation and retreat. In the context of ongoing climate warming, the moraine layer on glacier tongues is expected to further increase in thickness, thereby enhancing the stability of the glacier termini and reducing their sensitivity to climate change. Nonetheless, this “terminal stability” does not imply an overall improvement in glacier health. In the middle and upper reaches, new thin-layer moraine may be forming, or the ablation rates of exposed ice may be accelerating. These processes together contribute to sustained mass loss and thinning of the glaciers as a whole. The findings of this study also confirm that the Indus Basin has the largest glacier moraine coverage area and that the extent of moraine at glacier termini has shown an increasing trend during the study period. It should be noted, however, that the methodology adopted for moraine identification still faces certain challenges in thickly mantled glacier terminal regions. Accordingly, quantitative analysis of area changes primarily focuses on regions with a thin moraine cover. In these areas, the heat absorption effect of thin moraine layers intensifies glacier ablation, leading to a declining trend in glacier area.

**Figure 3:**
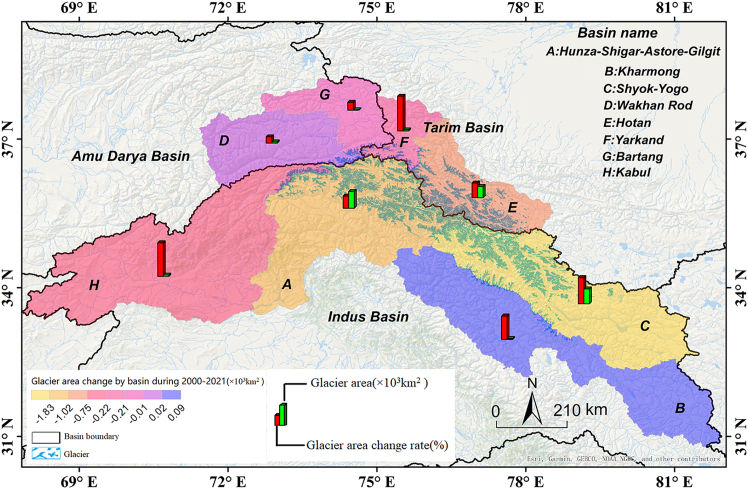
Glacier area change rates in the Karakoram basin from 2000 to 2021

We also calculated glacier area changes in 8 subbasins (Figure 3 and Table 2), namely, Hunza-Shigar-Astore-Gilgit (A), Kharmong (B), Shyok-Yogo (C), Wakhan Rod (D), Hotan (E), Yarkand (F), Bartang (G), and Kabul (H). In 2000 or 2021, the glacier area varied between 0.2 × 10^3^ and 0.75 × 10^3^ km^2^ in the Wakhan Rod, Yarkand, and Bartang subbasins. Moreover, from 2000 to 2021, the glacier area in the Wakhan Rod subbasin increased by 0.02 × 10^3^ ± 0.02 × 10^3^ km^2^, while in the other subbasin, the glacier area decreased by 0.01 × 10^3^ to 0.75 × 10^3^ km^2^. In general, from 2000 to 2021, the glacier area change ratio ranged from 5% to 30%, and the annual average value was lower than 1.5%. Specifically, the glacier area change ratio and annual average values were found to be maximum in the Yarkand subbasin (29% and 1%/yr, respectively), followed by the Hotan (12% and 0.59%/yr, respectively) and Bartang subbasins (6% and 0.32%/yr, respectively).

**Table 2:** Glacier area and its changes in the 8 subbasins in Karakoram from 2000 to 2021

Subbasins	Area (×10^3^ km^2^)		Area change (×10^3^ km^2^)	Area change rate (%)	Area change rate of year (%/yr)	Basin
	2000	2021				
A Hunza-Shigar-Astore-Gilgit	9.74 ± 0.29	8.72 ± 0.26	−1.02 ± 0.39	10	0.49	Indus
B Kharmong	0.47 ± 0.01	0.56 ± 0.02	+0.09 ± 0.02	19	0.95	
C Shyok-Yogo	8.19 ± 0.25	6.35 ± 0.19	−1.83 ± 0.31	22	1	
H Kabul	0.78 ± 0.02	0.56 ± 0.02	−0.22 ± 0.03	28	1	
E Hotan	5.98 ± 0.18	5.23 ± 0.16	−0.75 ± 0.24	12	0.59	Tarim
F Yarkand	0.71 ± 0.02	0.49 ± 0.01	−0.21 ± 0.03	29	1	
G Bartang	0.21 ± 0.01	0.19 ± 0.01	−0.01 ± 0.01	6	0.32	Amu Darya
D Wakhan-Rod	0.44 ± 0.01	0.46 ± 0.01	+0.02 ± 0.02	5	0.26	

The unit of the glacier area and its uncertainty is 10^3^ km^2^; “+” denotes an increase, and “−” denotes a decrease.

## Discussion

### Accuracy assessment for glacier identification

#### True sample selection

**Figure 4:**
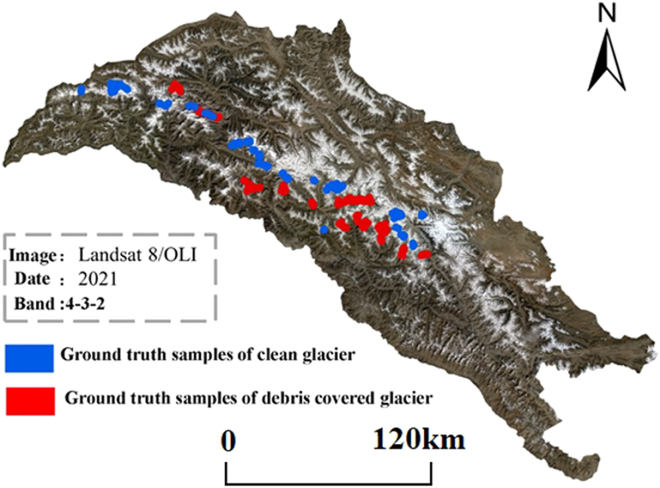
Distribution of true samples of clean glaciers and debris-covered glaciers in the KM region

The study of debris-covered glaciers poses a significant challenge in glacier research, necessitating the integration of surface temperature, TVDI, and the slope characteristics of glacier areas. High-spatial resolution optical remote sensing images from Google Earth, which represent an amalgamation of data from various sources, as well as Landsat-series remote sensing images, were employed for manual visual interpretation to establish ground truth samples for accuracy assessment. The specific procedure involved interpreting samples of clean ice and debris-covered glaciers, with 300 samples for each surface type (sample vectors representing pixels, with a resolution of 30 m), thereby ensuring a uniform and representative spatial distribution of the samples (Figure 4). In regard to clean ice samples, not only bare ice areas at the top of glaciers but also bare ice areas mixed with debris-covered glaciers were chosen. When selecting debris-covered glacier samples, strict reference was made to satellite images from Google Earth to avoid selecting surfaces with spectra similar to those of debris (such as bare soil). In addition, debris samples distributed on sunny and shady slopes were considered. Therefore, the selection of validation samples was representative and met the requirements for validation.

### Overall accuracy for debris-covered glaciers

The overall accuracy of debris-covered glacier classification was 67% and 71% in 2000 and 2021, respectively. Table 3 provides the accuracy of Karakoram glacier identification in 2000 and 2021, as measured by the overall accuracy, misclassification rate, and omission rate. To better demonstrate the effectiveness of glacier identification, the glacial debris extraction process and the glacial debris identification results for the Yashkuk Yaz Glacier and Baltoro Glacier are shown as examples in Figure 4.

**Table 3:** Accuracy ratios and error rates of debris-covered ice extraction for the abovementioned surface features

Year	LST	TVDI	Slope	L-T	L-T-S	Final result	Accuracy index	
2000	64	97	90	72	87	67	overall accuracy (%)	debris-covered glacier
2021	90	94	84	89	74	71		
2000	10	59	67	2	11	0	misclassification rate (%)	
2021	32	42	55	20	15	0		
2000	35	2	10	27	12	32	omission rate (%)	
2021	9	5	15	10	25	28		
2000	98	6	22	98	85	98	overall accuracy (%)	clean glacier
2021	98	45	38	98	98	98		
2000	82	56	45	84	85	82	overall accuracy (%)	both (clean glacier and debris-covered glaciers)
2021	75	68	55	83	81	85		

L-T, results for the LST and TVDI features; L-T-S, results for the LST, TVDI, and slope features; final result, final result after manual optimization.

With the assistance of semiautomated classification methods, only a small amount of fragmented misclassifications of small glaciers were needed to be manually removed. As the various features introduced in the debris-covered glacier identification process were combined, the misclassification rate gradually decreased, with the identification accuracy for debris-covered glaciers in both 2000 and 2021 exceeding 67%. The accuracy of clean glacier classification exceeded 98%, and the final classification accuracy exceeded 82%.

**Figure 5:**
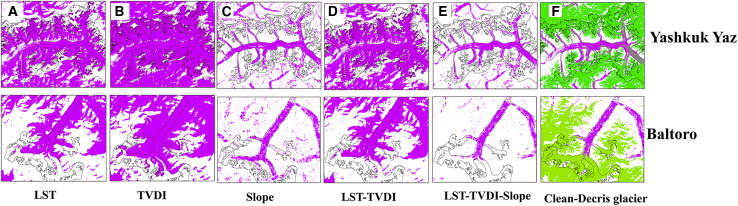
Schematic of the debris-covered glacier extraction process (examples: Baltoro Glacier and Yashkuk Yaz Glacier) (A) Surface temperature characteristic results. (B) Humidity characteristic results. (C) Slope characteristic results. (D) Surface temperature-humidity extraction results. (E) Surface temperature-humidity slope extraction results. (F) Final clean debris-covered glacier classification results.

Initially, the analysis of surface temperature characteristics in 2021 revealed the ability to extract 90% of glacier debris, albeit with a misclassification rate of 32% and an omission rate of 9%, as indicated in Table 3. In the process of using surface temperature characteristics to extract debris-covered glaciers, other surfaces with lower temperatures within the same threshold range were also identified (mainly water bodies, some vegetation, and bare rocks between elevations of 2,755 and 5,711 m) (Figure 5A). Second, the TVDI was used to extract 94.98% of debris-covered glaciers, with a misclassification rate of 42% and an omission rate of 5%. In addition to effectively extracting debris-covered glaciers, this feature enabled the extraction of water bodies, riverbeds, and partially clean glaciers with relatively high moisture contents (Figure 5B). Third, the slope feature was employed to extract 84% of debris-covered glaciers, with a misclassification rate of 55% and an omission rate of 15%. In addition to extracting debris-covered glaciers well, the slope feature enabled the classification of some water bodies (with more sediment) and most vegetation-covered areas (Figure 5C). Following the extraction of the aforementioned three features, the intersection between the surface temperature and TVDI, denoted as the L-T feature, was computed first. This intersection computation was executed utilizing the Raster Calculator tool within ARCGIS 10.3, with the model entailing the multiplication of the two features to derive the intersecting segment. Adopting 2021 as an example, the above process enabled the extraction of 89% of debris-covered glaciers, with a misclassification rate of 20% and an omission rate of 10%. Via the L-T feature, most areas with relatively high moisture contents, such as clean glaciers, were removed (Figure 5D). Second, the intersection between the L-T and slope features (referred to as the L-T-S feature: L-T feature ⊆ slope feature) was calculated. This process enabled the extraction of 74% of debris-covered glaciers, with a misclassification rate of 15% and an omission rate of 25%. Via the L-T-S feature, regions characterized by lower temperatures and steeper slopes were effectively eliminated, yet residual water bodies and sand dune surfaces persisted (Figure 5E). Clean glaciers meeting the specified threshold criteria were consistently included in the extraction process. Hence, it was necessary to manually optimize the final L-T-S feature to remove clean glaciers identified with both the L-T-S and TVDI features, particularly those with elevated moisture contents detected in the debris extraction process. Following optimization, the misclassification rate for debris identification was reduced to 0%, whereas the omission rate reached 28%. Via the use of false-color composite remote sensing images of the research area, the misclassified areas of other surfaces were subsequently eliminated, resulting in an identification rate for debris-covered glaciers of 71% after optimization (Figure 4F).

### Overall accuracy for clean glaciers and final mapping of glaciers

The overall accuracies of the glacier classification results for 2000 and 2021 were 82% and 85%, respectively (Table 3), and the overall accuracies of the clean glacier classification results were 98% and 98%, respectively. By comprehensively evaluating the overall accuracy of debris-covered glacier identification, we found that the final glacier identification accuracy (Figure 5F) was better than that of the results obtained from the other features, and the accuracy satisfied the requirements for subsequent analysis.

**Figure 6:**
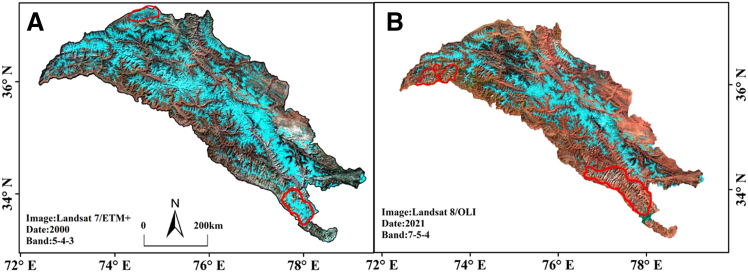
Karakoram false color composite remote sensing images (A) Image from 2000. (B) Image from 2021. Red polygons in the images denote areas covered by snow and fragmented clouds. In (A), the red polygon is mainly covered by snow, whereas in (B), the red polygon is largely covered by fragmented clouds.

To achieve high glacier identification accuracy, the quality of the original remote sensing images was strictly controlled, ensuring that the solar elevation angle of the original remote sensing images was greater than 30°. Images with minimal snow cover and cloud coverage in the glacier areas were selected to minimize uncertainty caused by snow, clouds, and shadows. Despite these efforts, the availability of Landsat images from 2000 was limited, and the image quality was relatively low, with some glacier areas covered by snow (Figure 6A), which affected the identification results. The quality of the original remote sensing images from 2021 was relatively satisfactory, but some glacier areas were partially covered by fragmented clouds (Figure 6B), which affected the identification accuracy.

The Landsat-simulated surface temperature and the TVDI were employed in conjunction with the ASTER-computed terrain slope to establish three classification features. These features were then integrated with contour data to generate contour-feature profiles, which were employed to delineate the boundaries of debris-covered ice.

### Glacier classification

**Figure 7:**
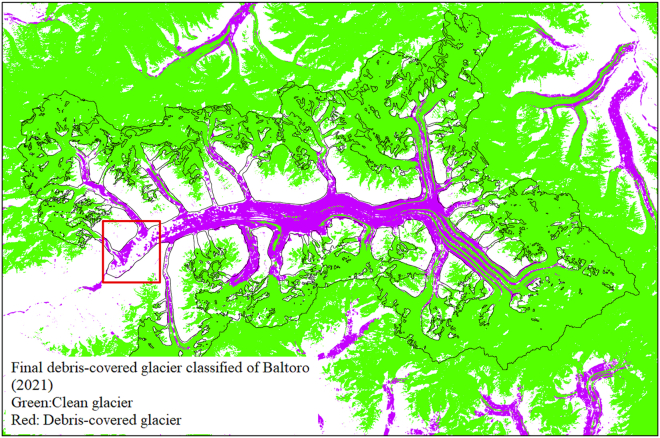
Classification results for the Baltoro Glacier (red squares denote areas with debris thicknesses above 1 m)

With the assistance of semiautomated classification methods, only a small amount of fragmented misclassifications of small glaciers were needed to be manually removed. As the various features introduced in the debris-covered glacier identification process were combined, the misclassification rate gradually decreased (Table 3). Additionally, the analysis results revealed significant differences in the TVDI feature in the identification process for debris-covered glaciers with different orientations. Glaciers located on the northern slopes of mountains, such as the Yashkuk Yaz Glacier, exhibit lower debris humidities and occur primarily in a frozen state. In contrast, glaciers on the southern slopes of mountains, such as the Baltoro Glacier, exhibit higher debris humidity, indicating a melting state and a greater presence of surface water. Therefore, when determining thresholds, it is necessary to consider the orientation of glaciers. The surface temperature and slope parameters showed minimal differences in the threshold values for debris-covered glaciers with different orientations. A comparison of the fully automated computer classification results with the semiautomated classification results involving manual vectorization intervention indicated that the former generally exhibited lower accuracy. Nevertheless, the implementation of manual vectorization methods is less efficient and demands greater effort, rendering it suitable primarily for individual glaciers or those within a limited geographical area. For large-scale and extensive glaciers, fully automated computer classification methods remain the ideal choice. Furthermore, the developed debris-covered glacier extraction methods still could not provide the identification of parts of glaciers with significant debris accumulation at their termini. As shown in Figure 7, the debris thickness in the red square region exceeds 1 m, whereas the other identified areas demonstrate debris thicknesses less than 1 m, with most debris thicknesses ranging from 0.2 to 0.5 m.^30^ The results indicated that the aforementioned methods can be used to effectively identify debris-covered glaciers with thicknesses less than 1 m. Previous studies have also revealed that glaciers with high debris cover are more challenging to identify.^36^

### Comparison with previous studies

Because debris-covered glacier is spectrally similar to the surrounding nonglacial rocks and land surface, it is crucial to develop novel algorithms for supraglacial debris identification. Previous studies have shown that debris-covered ice is generally accompanied by unique and irregular textures and topographic characteristics.^36^^,^^37^ Therefore, numerous researchers have applied the thermal infrared band to recognize debris-covered ice areas. For example, ASTER thermal infrared imagery, terrain slopes and aspects derived from DEM products, solar elevation angles, surface temperatures, and NDVI data have been used to delineate debris-covered ice boundaries.^36^^,^^38^ Additionally, compared with manually delineated debris-covered ice areas, automatic debris-covered ice extraction methods, such as decision trees and texture analysis, generally overestimate debris-covered ice areas by 25% and 31%, respectively.^36^ The accuracy of debris-covered ice extraction can exceed 85% with manual delineation, which conforms to the classification standard of the United States Geological Survey (USGS). During the recognition of debris-covered ice boundaries, the spatial resolution and quality of remote sensing images largely determine the accuracy of debris-covered ice classification, and the main error and uncertainty sources are clouds, seasonal snow, and shadows.^38^ The season and surface type variations can affect debris-covered ice recognition.^39^ Finally, it is difficult to identify the boundaries of fragmentary debris-covered patches.^38^ Xie et al.^17^ delineated glacier boundaries in the KM region since 1990, with their study area mainly focusing on the concentrated glacier clusters of the Karakoram. In contrast, the study area selected in this paper not only covers the main glacier concentration zones of the Karakoram but also includes the surrounding regions with scattered small glaciers. Generally, as glacier size increases, the relative proportion of debris-covered areas tends to decrease.^35^ Therefore, compared with the study by Xie et al.,^17^ the debris-covered glacier proportion obtained in this paper is slightly smaller, as a result of the larger study area.

In Karakoram, the aforementioned difficulties and potential uncertainty regarding debris-covered ice extraction also exist. We determined that, depending on individual classification features, favorable categorized results cannot be obtained and that the associated erroneous classification ratio is comparatively high. With the combination of three classification features, i.e., surface temperature, terrain slope, and humidity (TVDI), the erroneous classification ratio decreased. In addition, compared with those associated with the surface temperature and terrain slope, the humidity feature resulted in large differences with respect to debris-covered ice recognition in different aspects. Thus, it is necessary to consider the terrain aspect when extracting debris-covered ice areas. The analysis results revealed the glaciers located on the northern slopes of mountains, such as the Yashkuk Yaz Glacier, exhibit lower debris humidities and occur primarily in a frozen state. In contrast, glaciers on the southern slopes of mountains, such as the Baltoro Glacier, exhibit higher debris humidity, indicating a melting state and a greater presence of surface water. Therefore, when determining thresholds, it is necessary to consider the orientation of glaciers. In comparison, in many cases, automatic extraction of glacierized regions is less accurate than manual delineation. However, manual delineation is a time-consuming task with a high workload and is solely suitable and applicable for glacier area extraction in small regions. To extract large-scale glacier areas (i.e., HMA), an automatic classification approach is the most suitable option. Consequently, a semiautomatic method was employed to extract glacier areas, with only a few small glaciers in the Karakoram subjected to artificial improvement in 2000 and 2021. We validated our glacier area results in 2000 with RGI v.6.0 data and found that the area was overestimated by 19%, which is better than that of previous automatic methods.^36^ Although the aforementioned methods for identifying debris-covered glacier can effectively detect supraglacial debris to a certain extent, it remains an important task to conduct ground validation of the identification results through field measurements in the future.

### Climate factor, light-absorbing particulates, and water vapor impact on ice ablation

Research indicates that temperatures in the KM region show an annual warming trend (with less pronounced warming in summer), annual precipitation is increasing but winter precipitation is decreasing, and increased cloud cover is leading to reduced terrestrial radiation flux. Under the combined influence of these climatic factors, glaciers in the Karakoram are tending toward a state of mass balance.^40^

Light-absorbing particulates (LAPs) on glacier surfaces effectively reduce glacier albedo, leading to increased absorption of solar radiation by the glacier surface. This, in turn, raises the surface temperature and accelerates glacier melt. The main types of LAPs include black carbon (BC), dust, and organic carbon (OC). In the Karakoram, the average concentration of BC in glaciers reported in BC research is approximately 50 ng g^−1^. According to the PKU and MIX^41^^,^^42^ emission inventories, the ground-level concentrations of BC in the KM region in April, July, and December were all below 0.6 μg/m^3^, which is considered a low range for BC. Studies have indicated that rising temperatures and decreasing snow accumulation are the dominant factors driving the decline in KM glacier albedo. While BC and dust have some influence, their contribution is relatively small. Therefore, in the context of global warming and in contrast to glacier retreat in other regions, the anomalous behavior of glaciers in the KM region may partly be attributed to the low concentration of LAPs in the region, resulting in a less pronounced impact on glacier albedo relative to other areas. The stability of ice surface albedo can reduce glacier loss.

When the content of aerosol particles in the atmosphere is high, the first impact is a change in local meteorological conditions, causing regional water vapor to form precipitation locally. This, in turn, affects the water vapor transported to the Tibetan Plateau. Increases and decreases in water vapor further influence glacier change, for example, the increase in local precipitation due to BC aerosols in the South Asian region, which reduces the water vapor transported to the plateau. Consequently, the Himalayan and Southeastern Tibetan glaciers affected by this water vapor deficit have been in a state of retreat.^43^

**Figure 8:**
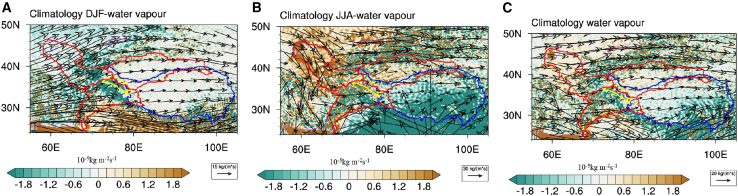
Water vapor trends in the Karakoram (A) Winter. (B) Summer. (C) Year. The red boundary is watershed basin, yellow boundary is Karakoram, and blue boundary is Tibetan plateau.

Figure 8 illustrates the pathways and changes in water vapor transport in the study area (data source: ERA5 monthly vertically integrated water vapor flux data with 0.25° resolution). As shown, water vapor in the KM region originates from three main directions: westerly winds bring water vapor from Central Asia and Europe along the Amu Darya River Basin. The Tarim River Basin primarily receives water vapor from the confluence of the westerly wind belt and monsoon circulation, while the surrounding mountains of the Tarim Basin partially block some water vapor. The Indus River Basin receives monsoonal water vapor from the southern Indian Ocean and the Arabian Sea. The monsoon primarily moves northward along the Himalayas, significantly impacting glacier replenishment in this basin.

According to water vapor trends of winter (Figure 8A), summer (Figure 8B), and year (Figure 8C) in Karakoram during 2000–2021, summer water vapor dominated the annual variation of water vapor in the region (Figures 8B and 8C). Compared with winter, more water vapor was transported to the glaciers in summer. Although this is the glacier melt season, the precipitation from water vapor transport plays a role in glacier accumulation to some extent. In winter, however, temperatures are inherently low, and even with less water vapor transport and weaker precipitation (Figure 8A), significant glacier melt is not promoted. Therefore, water vapor transport has a significant impact on Karakoram glacier changes. Examining the river basins, it was found that water vapor transport in the Tarim River Basin has not shown a significant decreasing trend (especially in summer) compared with the Indus River Basin and the Amu Darya River Basin, thus leading to increased precipitation that promotes regional glacier growth.

### Debris-covered glacier and surge-type glaciers’ impact on ice ablation

**Figure 9:**
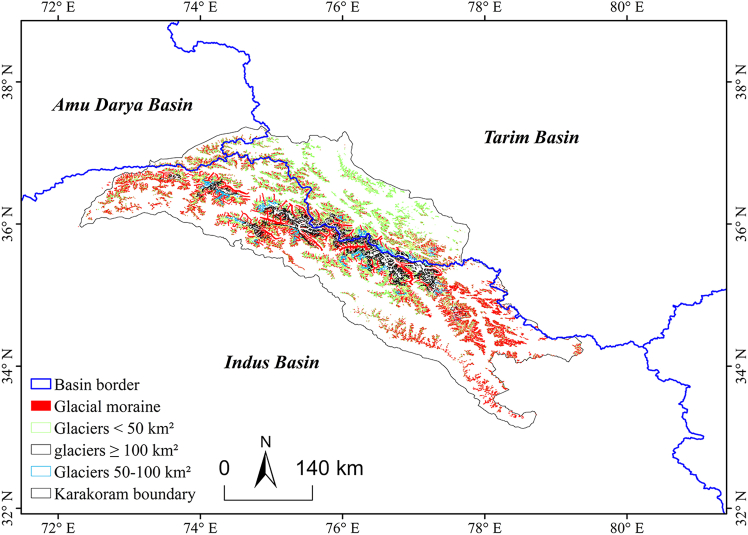
Supraglacial debris cover status on Karakoram glaciers of different sizes

Figure 9 illustrates the distribution of surface moraine coverage on glaciers across various basins in the KMs. Compared with the Amu Darya and Tarim River basins, the Indian River Basin has the largest area of small glaciers covered by surface moraines. Small glaciers, as a vital component of the cryosphere, change more rapidly than large glaciers, and changes in surface moraine coverage can more sensitively reflect the processes of glacial retreat and melting. Previous studies have shown a clear retreating trend in small glaciers in the KM region,^44^ suggesting that the thickness of surface moraines on these glaciers is insufficient to effectively block heat. Instead, due to heat absorption, they exacerbate glacial melting, thereby influencing changes in glacier area. Furthermore, research indicates that there are 208 surging glaciers in the Amu Darya, Tarim, and Indian River basins.^45^ Glacier surging can redistribute surface moraines and affect the overall extent and thickness of surface moraine coverage. Consequently, changes in the surface moraine coverage on glaciers are an important future research direction for glacial studies and hold significant implications for downstream water resource management.

### Limitations of the study

Glacier area is one of the key parameters reflecting changes in glaciers. The majority of glaciers in the KM are covered by supraglacial debris. Due to the spectral similarity between supraglacial debris and adjacent surface types (i.e., rock or soil), it is challenging to accurately delineate glaciers covered by debris. Traditional methods, such as band ratio and supervised classification, are not suitable for identifying debris-covered areas, which is one of the main challenges in studying glacier area changes in the KM region. In this study, building on previous research, we utilized Landsat remote sensing imagery, ASTER GDEM data, and a variety of surface features—including glacier reflectance, topography, surface temperature, and moisture—to comprehensively map the boundaries of debris-covered glaciers.

We adopted a semi-automatic classification approach to extract glacier extent in the KM region between 2000 and 2021 and analyzed the changes over time. For the two types of glacier surfaces—clean ice and debris-covered ice—different identification methods were employed: glacier boundaries in clean ice areas were primarily delineated using supervised classification based on Landsat imagery, while debris-covered glaciers were identified by integrating surface temperature and TVDI derived from Landsat imagery, along with slope data extracted from ASTER DEM products. After classification, the overall accuracy and misclassification rate were used to evaluate the results. The final overall classification accuracy exceeded 82%, with the accuracy for clean ice glaciers being >98% and that for debris-covered glaciers >67%. Table 3 details the classification accuracy of debris-covered glaciers when using single index parameters; most parameters showed overall accuracy of >80%, indicating that the selected data and indices are well suited for mountain glacier extraction.

Nevertheless, this study was still unable to effectively identify glaciers with thick supraglacial debris. Therefore, future research should focus on combining field observations and advanced techniques such as LiDAR or drone-based 3D reconstruction to analyze surface undulations and sediment morphology, aiming for more accurate identification of thick debris cover. In addition, further research is needed to deepen our understanding of the driving factors and processes of glacier changes in the KM.

## Resource availability

### Lead contact

Further information and requests for resources should be directed to and will be fulfilled by the lead contact, Drolma Lhakpa (lhakdron@yeah.net).

### Materials availability

All the materials of this study are available from the lead contact without restriction and upon reasonable request.

### Data and code availability

• Landsat and ASTER Data used in this study are available from the lead contact upon reasonable request.
• Raw Landsat data sources and the final results used in the article are available https://doi.org/10.5281/zenodo.17672697). Detailed information regarding all glacier identification steps is available from the lead contact upon request. The manual methods used in this study (based on the ARCGIS 10.3 and ENVI 5.3 platforms) do not involve any programming or coding.
• Additional information regarding the data and analyses presented in this study is available from the lead contact upon request.

## Acknowledgments

This work was supported by the 10.13039/501100018543Natural Science Foundation of Tibet Autonomous Region (grant no. XZ202501ZR0136), National Natural Science Foundation of China (grant no. 42061014), Major Science and Technology Project of the Xizang Autonomous Region (XZ202402ZD0006), the Youth Innovation Team of the 10.13039/501100002885China Meteorological Administration (Climate Change and its Impact on the Tibetan Plateau) (no. CMA2023QN16), the Second Tibetan Plateau Scientific Expedition and Research Program (STEP) (grant no. 2019QZKK0201), the Mêdog and Shigatse National Climatological Observatories, 10.13039/501100002885China Meteorological Administration (CMA). M.L. was supported by the National Natural Science Foundation of China (grant no. 42301151) and 10.13039/501100002858China Postdoctoral Science Foundation (grant no. 2022M712853). We are grateful to Chang-Qing Ke and Minghong Liu for their numerous comments on this study. We appreciate Yangzom Pema’s contributions during the revision process of the manuscript.

## Author contributions

D.L. conceived the study, wrote the manuscript, and performed data analysis; M.L. and B.C. reviewed the logic of the article; B.T. and T.Y. contributed to literature retrieval and management. All authors have read and agreed to the published version of the manuscript.

## Declaration of interests

The authors declare that they have no known competing financial interests or personal relationships that could have appeared to influence the work reported in this paper.

## STAR★Methods

### Key resources table

**Table:** 

REAGENT or RESOURCE	SOURCE	IDENTIFIER
**Deposited data**		

Data source and Karakoram glacier area data	Zenodo	https://doi.org/10.5281/zenodo.17672698

**Software and algorithms**		

ARCGIS10.3	Esri	https://www.esri.com
ENVI 5.3	Exelis Visual Information Solutions	https://envi.geoscene.cn/

### Experimental model and study participant details

Omitted as our study does not involve biological models.

### Method details

#### Data and methods

##### Landsat data

To minimize interference from seasonal snow cover and clouds in glacier identification, satellite images acquired from September to December under clear-sky conditions with minimal snowfall impacts were selected as annual glacier change reference data.

The obtained Landsat datasets were primarily employed for three purposes: (1) glacier surface temperature retrieval via thermal infrared bands, (2) TVDI calculation, and (3) clean glacier boundary identification via multispectral analysis.

##### ASTER GDEM v3

We utilized ASTER Global Digital Elevation Model version 3 (ASTER GDEM v3) products to derive the glacier slope within the KM. ASTER GDEM v3 offers a higher spatial resolution, with a horizontal accuracy of ±15 m and a vertical accuracy of ±15–25 m, compared with that of previous versions (v1 and v2). This product is sufficiently accurate for analysing the glacier surface morphology.^46^

##### Computing platform and data processing

We utilized ARCGIS 10.3 and ENVI 5.3 software for data processing and applied a semi-automatic classification method to generate glacier area data for the Karakoram Mountains in 2000 and 2021. The satellite remote sensing data used were sourced from Landsat-7 TM and Landsat-8 OLI imagery (30-meter spatial resolution, 16-day revisit cycle), which provide detailed surface reflectance information. Glacier surfaces were categorized into clean ice and debris-covered ice, with different methods employed for their identification: for clean ice areas, glacier boundaries were primarily delineated using supervised classification based on Landsat imagery. For debris-covered ice, a combination of surface temperature and Temperature-Vegetation Dryness Index (TVDI) derived from Landsat imagery, along with topographic slope extracted from the ASTER Digital Elevation Model, was utilized. The evaluation process also considered metrics such as overall accuracy and misclassification rates.

### Quantification and statistical analysis

The overall accuracy of glacier recognition was 82% and 85% in 2000 and 2021, respectively. In 2000, the overall accuracy and misclassification ratio of debris-covered ice delineation obtained from the surface temperature feature were 64% and 10%, respectively. Compared with those obtained with the surface temperature feature, the accuracy and misclassification ratio of debris-covered ice delineation derived from the TVDI (>90% and >59%) and slope features (>90% and >59%) were greater. In 2021, the overall accuracy and misclassification ratio of de-bris-covered ice delineation derived from the surface temperature, TVDI, and slope features were >84% and >32%, re-spectively. When the three recognition features were combined, the de-bris-covered ice accuracy was above 72%, and the misclassification ratio was below 20%. The details are as follows.

#### Glacier extraction

A semi-automated classification method was used to identify glaciers in the Karakoram and analyze their spatiotemporal changes. Clean glaciers from 2000 to 2021 were extracted using supervised classification based on Landsat images. Glacier debris cover for 2000 and 2021 was identified using surface temperature and humidity data derived from Landsat images and slope features calculated from ASTER data. By combining the classified clean glaciers and debris-covered glaciers, the classification results were evaluated using overall accuracy and misclassification rate. Furthermore, glacier area changes in the Karakoram region and different basins from 2000 to 2021 were analyzed. The method refers to Flowchart (Figure S1). All data processing was conducted using ARCGIS 10.3 and ENVI 5.3 software.

#### Clean glacier classification

The Maximum Likelihood Classification (MLC) method was employed to extract clean ice boundaries. When choosing glacier samples for analysis, the use of high-resolution remote sensing images from Google Earth to verify the purity and cleanliness of the selected glacier areas is recommended. Via the use of supervised classification, more than 100 clean glacier samples were obtained. The classified clean glaciers demonstrated a high level of agreement with the original images, except for the debris-covered glaciers (Figure S2).

#### Mapping of debris-covered glaciers

Glacier surfaces were classified into two types: debris-covered and clean ice zones. For debris-covered ice extraction, we employed three features, namely, the surface temperature, TVDI, and terrain slope. The former two features were derived from Landsat imagery, and the last feature was computed from ASTER GDEM v3. Additionally, we assessed the accuracy of debris-covered ice extraction and calculated the uncertainty in the corresponding boundaries. A supervised classification method was utilized for delineating clean ice. The boundaries of debris-covered ice and clean ice were subsequently merged to determine the total glacier area in the Karakoram region in 2000 and 2021.

#### Extraction of land surface temperature (LST) data

Debris-covered glaciers pose significant challenges in boundary delineation because of their spectral similarity with rock and sediment surfaces. However, their distinct thermal characteristics compared with those of the surrounding terrain provide an effective identification criterion. Studies have demonstrated a positive correlation between the debris surface temperature and thickness,^39^^,^^47^ with temperatures increasing at lower elevations and with terminus areas typically exhibiting higher temperatures than those in upper zones^36^^,^^48^^,^^49^^,^^50^; moreover, substantial melt suppression occurs when the debris thickness exceeds 0.3–0.4 m.^36^^,^^47^^,^^50^^,^^51^^,^^52^ These thermal signatures have enabled glacier dynamics monitoring in the southeastern part of the Tibetan Plateau,^48^ and serve as critical parameters for ablation quantification.^30^

Atmospherically corrected LST data were employed for glacier mapping. In the atmospheric correction process, key parameters (atmospheric transmittance and upwelling/downwelling radiance) derived from National Aeronautics and Space Administration (NASA) platforms were incorporated (Table). In the algorithm, thermal radiative transfer equations are employed to eliminate atmospheric interference, with band 6 (Landsat 7) and band 10 (Landsat 8) selected as the optimal thermal infrared channels. A complete processing workflow comprising radiometric calibration of multispectral data and atmospheric correction of thermal bands was implemented in ENVI 5.3 software.

Assuming that the surface is a Lambertian body and neglecting the energy from solar radiation, the thermal infrared radiation transfer equation can be expressed as follows:

(Equation 1) *L*_*λ*_ = [*ℇB*(*T*_*s*_)+(1-*ℇ*)*L*^↓^)]*τ*+*L*^↑^

where L*λ* is the radiance at the top of the atmosphere (TOA), which is the radiation measured by the sensor; *B*(*Ts*) is the blackbody radiance derived from Planck’s law (unit: W/(m^2^·sr·μm)); *Ts* is the surface temperature (unit: K); ℇ is the surface emissivity; *τ* is the atmospheric transmittance; and *L*↑ and *L*↓ denote the atmospheric upwelling radiance and downwelling radiance, respectively (unit: W/(m^2^·sr·μm)).

On the basis of Equation 1, *B*(*Ts*) can be calculated as follows:

(Equation 2) *B*(*T*_*s*_) = [*L*_*λ*_-*L*^↑^-*τ*(1-*ℇ*)*L*^↓^)]/*τℇ*

Finally, the surface temperature can be obtained through the inverse operation of the Planck function:

(Equation 3) $$T_{s}=K_{2}/\ln\left(\frac{K_{1}}{B\left(T_{s}\right)}+1\right)$$

where K1 and K2 are radiation constants. In different Landsat series, different K1 and K2 values are used for surface temperature inversion. For the TM, K1 = 607.76 W/(m^2^·sr·μm), and K2 = 1260.56 K; for the ETM+, K1 = 666.09 W/(m^2^·sr·μm), and K2 = 1282.71 K; for the Thermal Infrared Sensor (TIRS), K1 = 774.89 W/(m^2^·sr·μm), and K2 = 1321.08 K.

According to the above, Ts calculation requires first the calculation of B(Ts), and τ, L↑, L↓, and ε are important parameters for obtaining B(Ts). Moreover, τ, L↑, and L↓ can be calculated using the Atmospheric Correction Parameter Calculator provided by NASA (https://atmcorr.gsfc.nasa.gov/).^53^^,^^54^ The calculated parameters are listed in Table. This tool relies on global atmospheric profile data simulated by the National Centers for Environmental Prediction and the Moderate Resolution Atmospheric Transmission (MODTRAN) model. The MODTRAN model aims to simulate the radiative transfer process between the surface and the sensor, thereby outputting τ, L↑, and L↓.

**Table:** Atmospheric parameters needed for surface temperature inversion

Line/row number	Lon.	Lat.	Atmospheric transmissivity in the thermal infrared band (T)		Upwelling radiance (L↑)		Downwelling radiance (L↓)		Note
			2000	2021	2000	2021	2000	2021	Due to the lack of surface-related parameters, the calculation results are obtained via the radiative transfer equation (RTE)
14636	78.15	35.65	0.97	0.98	0.15	0.10	0.26	0.19	
14637	77.76	34.22	0.97	0.98	0.13	0.09	0.23	0.16	
14735	76.97	37.08	0.94	0.92	0.34	0.56	0.59	0.97	
14736	76.70	35.67	0.99	0.93	0.06	0.41	0.11	0.71	
14737	76.32	34.23	0.98	0.92	0.09	0.55	0.15	0.96	
14835	75.53	37.10	0.94	0.89	0.28	0.78	0.48	1.34	
14836	75.13	35.66	0.97	0.94	0.14	0.35	0.25	0.61	
14934	74.35	38.53	0.97	0.98	0.18	0.10	0.32	0.18	
14935	73.94	37.09	0.97	0.96	0.13	0.27	0.22	0.48	
15034	72.77	38.52	0.98	0.97	0.09	0.16	0.16	0.29	
15035	37.09	72.37	0.97	0.97	0.16	0.18	0.27	0.32	
15135	70.79	37.08	0.98	0.93	0.10	0.58	0.18	1.00	

To determine ε, it is necessary to first calculate the normalized difference vegetation index (NDVI) to estimate the Percentage Vegetation coverage (PV) and ε. We can calculate the NDVI via Equation 4:

(Equation 4) *NDVI*=(*NIR*-*R*)/(*NIR*+*R*)

where NIR denotes the near-infrared band, which is derived from band 5 of Landsat-8 and band 4 of Landsat-7. Moreover, R is the red band, which is derived from band 4 of Landsat-8 and band 3 of Landsat-7. The vegetation coverage (PV) can be estimated using the pixel-based binary model given below:

(Equation 5) *PV*= (*NDVI*-*NDVIsoil*)/(*NDVIveg*-*NDVIsoil*)

where NDVIsoil denotes the NDVI value of pure bare soil or vegetation-free pixels, and NDVIveg is the NDVI value of pure vegetation pixels. Notably, NDVIsoil is the minimum NDVI value of vegetation, and NDVIveg is the vegetation NDVI value corresponding to a cumulative frequency of 95%. Sobrino et al. (2004)^55^ considered 49 soil spectra and obtained an average soil emissivity of 0.973 (with a standard deviation of 0.004). Combining this value with a vegetation emissivity of 0.99, ℇ can be calculated as follows:

(Equation 6) *ℇ*=0.04×*PV*+0.986

Taking an image of the study area on September 6, 2021 as an example, Figure 3 shows the procedure of LST inversion. Figure S3A shows the outcome following atmospheric and radiometric corrections, while Figure S3B shows the NDVI results. The NDVI was derived from vegetation spectra by calculating the ratio of the difference between the near-infrared and red band reflectance values to their sum, which is based on the characteristic high reflectance in the near-infrared band and low reflectance in the red band. Figure S3C shows the blackbody radiance, which ranges from 3.54 to 13.98 W/(m^2^·sr·μm), with notably lower levels observed in glacier regions. Figures S3D and S3E show the LST and glacier surface temperature, respectively, across the entire region. The nonglacier areas exhibited elevated LST values, peaking at 50°C. The Karakoram region experiences intense solar radiation and notable vertical climate differences attributed to its extensive topographical variations. In contrast to larger areas, the Karakoram region exhibits a lower rate of heat dissipation, which indicates the storage of a large amount of heat at the surface, resulting in higher LST values in nonglacier areas. The glacier surface temperature mainly ranged from −10°C to −2.5°C, with an average temperature of −5.01°C (Figure S3F). The highest glacier surface temperature was 15.16°C, which occurred mainly in moraine areas (Figure S3E), primarily because of heat absorption by dark-coloured moraines.

The aforementioned calculations were conducted for all 26 images from both 2000 and 2021 encompassing the study area, resulting in the compilation of surface temperature data for each year via stitching. The NDVI was used to derive the TVDI. The maximum and minimum surface temperatures in 2021 were higher than those in 2000, and the area with low temperatures decreased (Figures S4A and S4B). The surface temperature in nonglacier areas was significantly higher than the glacier surface temperature, with the latter not exceeding 6°C (2000: 5.72°C; 2021: 4.99°C) (Figures S4A–S4D). In 2000, the glacier surface temperature mainly ranged from −20°C to −10°C, with a peak distribution of approximately −16.5°C to −16°C (Figure S4E). In 2021, the peak distribution of the surface temperature shifted to the right, ranging from −15.5°C to −15°C, and the average temperature increased by 1.51°C compared with that in 2000 (Figure S4F). The selected time range was strictly controlled to autumn months (September to November), with clear skies and no snow cover on the glaciers. Within this limited range of images, the quality of the Landsat series remote sensing images obtained for 2000 was relatively poor, and there was a small amount of snow cover on the glaciers and surrounding surfaces, which reduced the glacier surface temperature. This resulted in lower surface temperatures within the glacier area in 2000. However, the control effect of glacial moraines on the surface temperature cannot be neglected.

To facilitate the comparison of surface temperature features with other features, we standardized the surface temperature and extracted it within specific transverse sections (Figure S5). Three transverse sections (zone 3, representing the glacier terminus; zone 2, representing the middle of the glacier; and zone 1, representing the peak of the glacier) were chosen for surface temperature extraction. The results revealed that zone 3 mainly comprised clean ice and rock-fragment ice, with mean normalized LST values for clean ice, rock-fragment ice, and rock of 0.30, 0.40, and 0.64, respectively. Zone 2 largely comprised debris-covered ice, and the mean normalized LST values for debris-covered ice, rock-fragment ice, and rock were 0.50, 0.40, and 0.67, respectively. Zone 1 was primarily covered by debris-covered ice, and the mean normalized LST varied between 0.67 and 0.74, which is lower than that of the adjacent rock (0.90). Therefore, the mean normalized LST values for debris-covered ice and the adjacent rock surface at the glacier terminus are typically higher than those at the middle and peak of the glaciers. As a result, we selected surface types with mean normalized LST values ranging from 0.50 to 0.74 for debris-covered ice classification.

#### TVDI retrieval based on Landsat imagery

Sandholt et al. (2002)^56^ employed the LST and NDVI to calculate the TVDI, a metric utilized for estimating the surface moisture content. Notably, a higher TVDI value indicates a lower moisture content. The TVDI was computed using the TVDI model tool in ENVI 5.3 software. The TVDI can be computed via Equations 7, 8, and 9.

(Equation 7) $$TVDI=\frac{T_{S}-T_{Smin}}{T_{Smax}-T_{Smin}}$$

(Equation 8) *T*_*Smax*_ = *a*_1_+*b*_1_×*NDVI*

(Equation 9) *T*_*Smin*_ = *a*_2_+*b*_2_×*NDVI*

where *T*_*S*_ is the LST, *T*_*Smin*_ denotes the minimum LST for the same NDVI, *T*_*Smax*_ is the maximum LST for the same NDVI, and *a*_1_, *a*_2_, *b*_1_ and *b*_2_ are coefficients of the dry- and wet-side linear-fitting equations.

In general, the TVDI served as a reliable indicator for distinguishing between clean ice, debris-covered ice, and other surface types, such as rock. Given the significant variations in the TVDI values among different debris-covered ice surfaces, we selected two representative glaciers (Yashkuk Yaz Glacier and Baltoro Glacier) for TVDI calculation. These two glaciers were selected because of their contrasting ice cover characteristics: the Yashkuk Yaz Glacier is entirely covered by debris and is located on the shaded side (Figures S6A and S6B), whereas the Baltoro Glacier features a mix of debris-covered and clean ice zones and is located on the sunny side (Figures S7A and S7B). These glaciers are the two main glacier types in Karakoram.

For the Yashkuk Yaz Glacier a polygon with a pixel counts greater than 10000 was delineated across the surface of the glacier covered with debris (S 6b), and debris-covered ice was subsequently clipped on the basis of this polygon. The mean TVDI was then calculated within each 30 × 30 moving window. The results revealed that the TVDI of the Yashkuk Yaz Glacier mostly ranged from 0.73–0.84 (Figure S6C), which indicates that the moisture content is low and marginally differs from those of other surface types (i.e., rock). Compared with that of the Yashkuk Yaz Glacier, the TVDI of the Baltoro Glacier was lower, ranging from 0.50 to 0.64, indicating a higher moisture content. This difference in TVDI values between the two glaciers could be attributed to the higher ice content in debris-covered ice of the Baltoro Glacier.

For the Baltoro Glacier, In order to determine the TVDI threshold for supraglacial debris-covered glaciers, cross-sections of the Baltoro Glacier and their corresponding TVDI values were first extracted. This approach allows the presentation of all surface feature values along a cross-section and hence the separation of TVDI values corresponding to debris-covered glaciers. As shown in the figure, these include the TVDI values of a clean glacier cross-section (S 7(c)), the TVDI values of a cross-section in a mixed debris-and-ice covered zone (Figure S7D), and the TVDI values of a cross-section purely covered by supraglacial debris or bare ground (Figure S7E). Due to the mixture of debris and ice on the surface of the Baltoro Glacier, there is relatively high moisture content in the supraglacial debris, with TVDI values ranging between 0.50 and 0.64.

The variation of TVDI values is mainly influenced by the moisture storage status of the supraglacial debris, the glacier’s geographical location, and the degree of ablation. The greater the amount of embedded ice within the debris, the higher the moisture content and the lower the TVDI value; conversely, with less moisture, the TVDI value tends to be higher. A comprehensive analysis of the TVDI values of supraglacial debris for glaciers in the region shows that the TVDI values of typical debris-covered glaciers selected in 2000 and 2021 did not exhibit significant fluctuations. After repeated testing and comprehensive comparison of the data, the TVDI threshold was finally determined to be 0.76–0.93 for the year 2000 and 0.75–0.94 for the year 2021.

The ASTER GDEM product was adopted to calculate the terrain slope in Karakoram (Figure S8). The terrain slope can serve as a suitable indicator for separating debris-covered ice from other surface types (i.e., rock). Overall, the terrain slope of debris-covered ice ranges from 0–12.42°. Previous studies have indicated that the terrain slope of debris-covered ice barely exceeds the range of 12–24°.^35^^,^^57^ To automatically extract debris-covered ice in Karakoram, we classified glaciers with surface terrain slopes ≤ 8° as debris-covered glaciers.

#### Uncertainty

To accurately assess the glacier coverage in the Karakoram region, Landsat images obtained after the ablation season with minimal cloud cover were selected to mitigate the impacts of seasonal snow and cloud cover. Additionally, optical images with a solar elevation angle greater than 30° were filtered. Despite the numerous preprocessing steps implemented, it remains essential to estimate the uncertainty in the determined glacier areas. A fixed glacier area uncertainty ratio of 3% was applied to evaluate potential misclassifications of glacier areas in the Karakoram region and its drainage basin in 2000 and 2021.^58^^,^^59^ The uncertainties in the obtained glacier area and its changes can be calculated with Equations 10 and 11, respectively.

(Equation 10) *σ*_*area*_ = *A*×*λ*

where *σ*_*area*_ denotes the uncertainty in the glacier area, *A* is the glacier area, and *λ* is the ratio, at 3%.

(Equation 11) $$U_{change}=\sqrt{U_{1}^{2}+U_{2}^{2}}$$

where *U*_*change*_ is the uncertainty in the glacier area, and *U*_*1*_ and *U*_*2*_ are the uncertainties in the glacier areas in 2000 and 2021, respectively.
