# Supplementary material for: Debris-covered glacier identification in the Karakoram aided by thermal remote sensing
Source: iScience. 2026 Mar 13;29(5):115148. doi: 10.1016/j.isci.2026.115148 (PMC13122822; doi:10.1016/j.isci.2026.115148)
Supplement: Document S1. Figures S1–S8 [file mmc1.pdf]

iScience, Volume *29*

Supplemental information

Debris-covered glacier identification in the Karakoram aided by thermal remote sensing

Drolma Lhakpa, Mengmeng Li, Basang Tsedan, Tsomo Yongqing, and Bin Cheng

Supplemental Figure

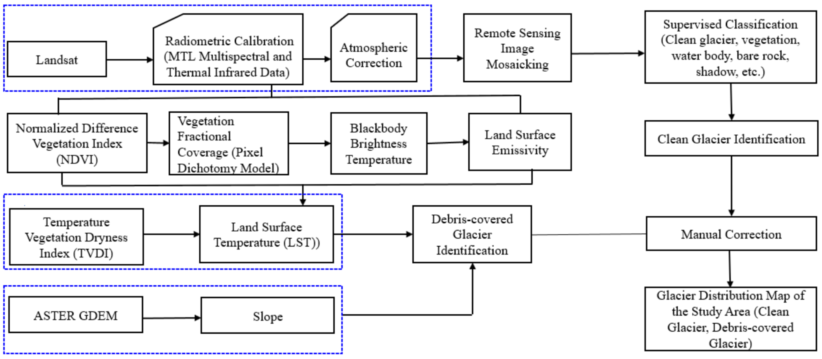

S1. Flowchart of Glacier Extraction

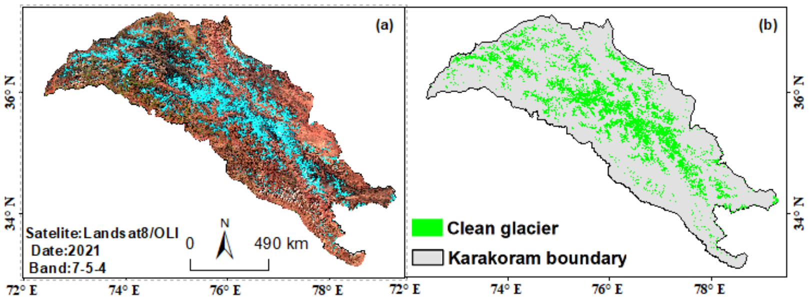

S2. Map for effective clean glacier extraction in Karakoram (remote sensing image (a); clean glacier extraction map (b))

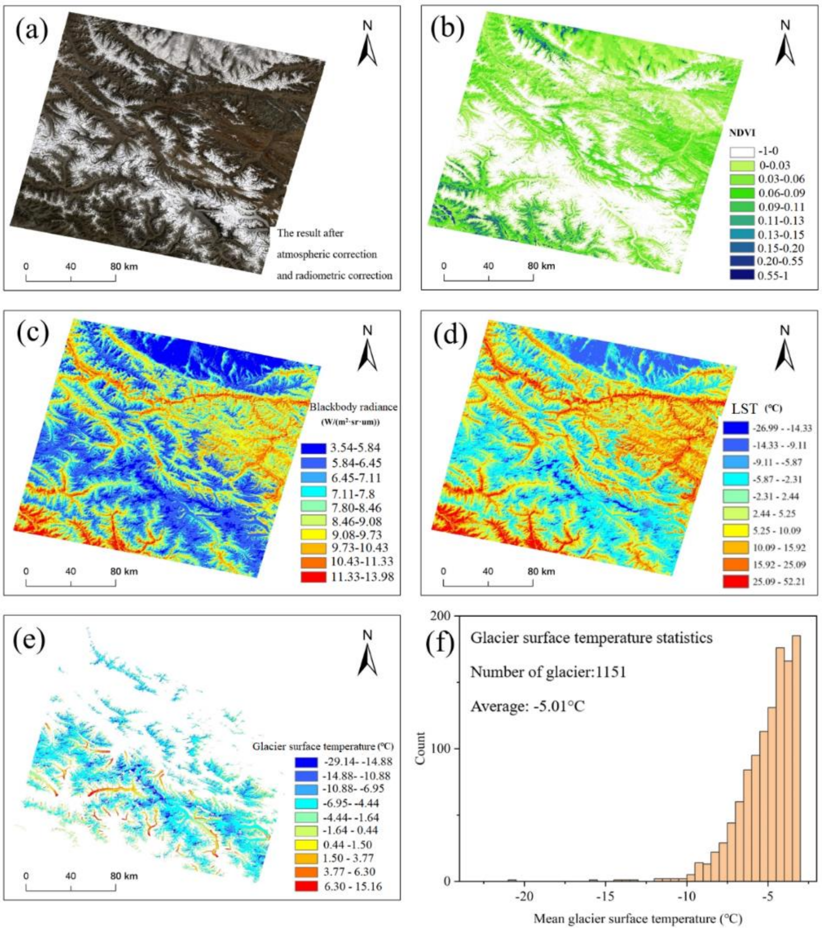

S3. True-colour composite image after atmospheric correction and radiometric calibration (example Landsat remote sensing image: Strip number 148/35), (b): NDVI, (c): blackbody radiance given by Planck's law, (d): land surface temperature, (e): glacier surface temperature and (f): distribution of the glacier surface temperature

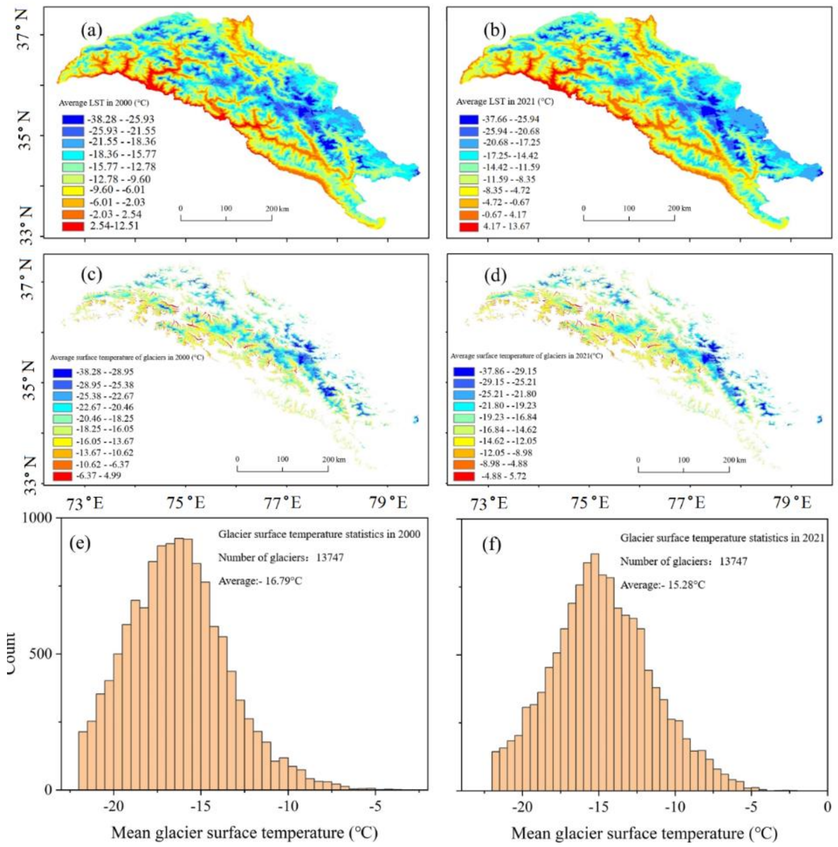

S4. Spatial distribution of the surface temperature in the Karakoram Mountains in 2000 (a) and 2021 (b); spatial distribution of the glacier surface temperature in the Karakoram Mountains in 2000 (c) and 2021 (d); distribution of the glacier surface temperature in 2000 (e) and 2021 (f)

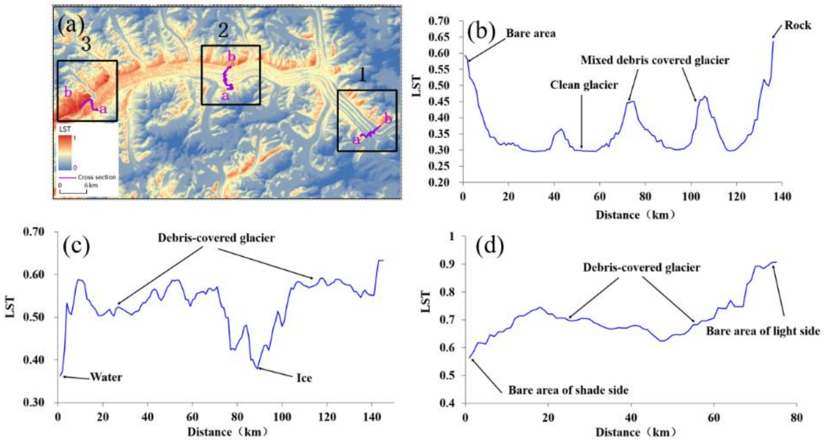

S5. LST features in the three selected glacier transverse sections (the sample glacier is the Baltoro Glacier in 2021). (a) Three selected transverse sections, which are denoted by purple lines (the background image is a Landsat-8 true-colour image). Surface temperature characteristic values corresponding to the cross-section of Baltoro Glacier in (b) Zone 1, (C) Zone 2, and (d) Zone 3.

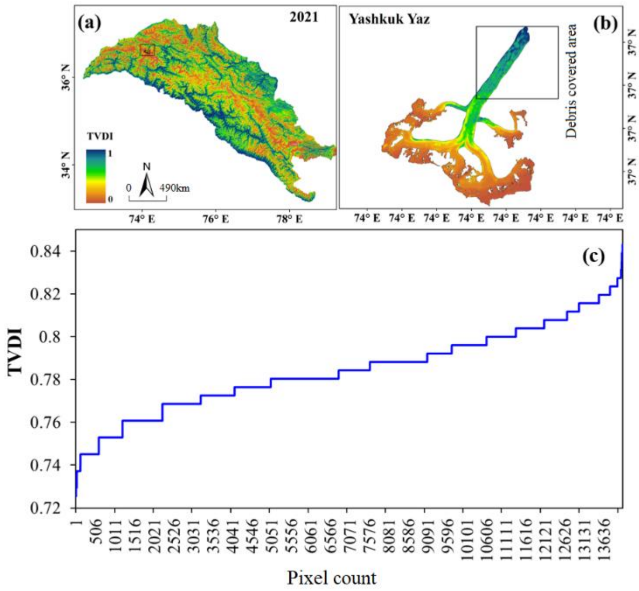

S6. Extraction of the glacier surface humidity based on the TVDI ((a): The TVDI map of karakoram, (b): The TVDI map of Yashkuk Yaz Glacier, (c): The TVDI values of each pixel at the end of Yashkuk Yaz Glacier (the region indicated by box in (b))

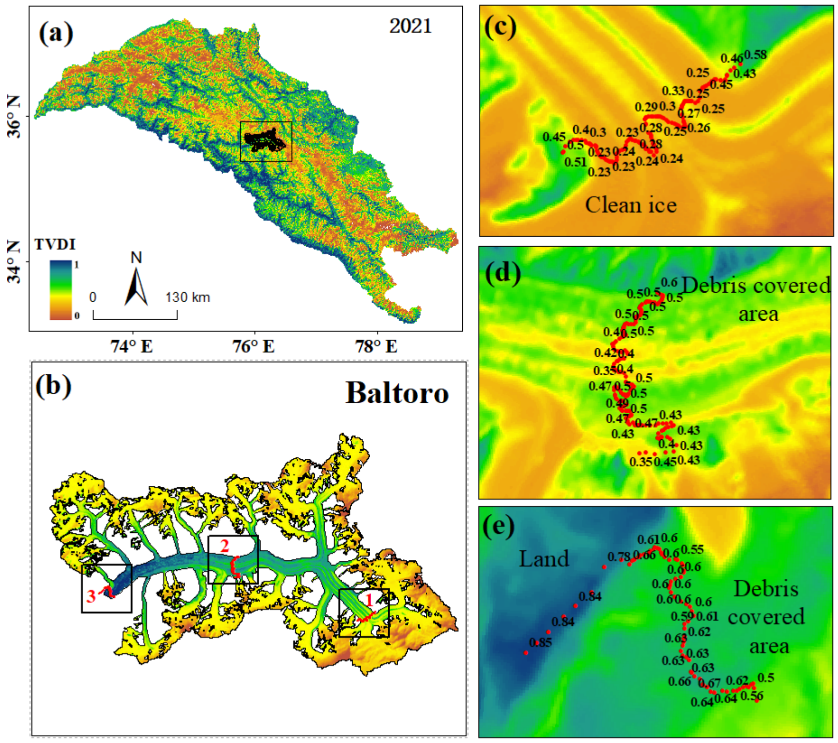

S7. Calculation of Baltoro glacier surface humidity based on TVDI. (a) The TVDI map of Karakoram, (b) The TVDI map of Baltoro Glacier, (c) TVDI values of the mid upper cross-section of the Baltoro Glacier (Cross section of Zone 1 in Figure b, (d) TVDI values of the middle cross-section of the Baltoro Glacier (Cross section of Zone 2 in Figure b, (e) TVDI values of the terminal cross-section of the Baltoro Glacier (Cross section of Zone 3 in (b))

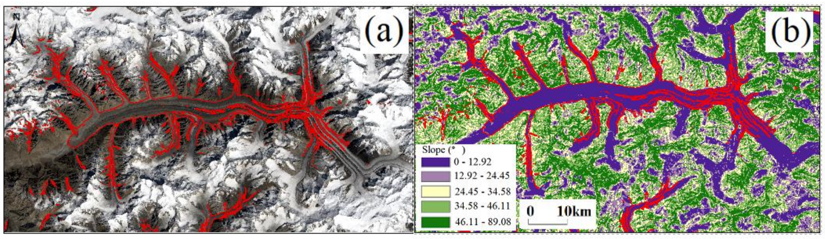

S8. Slope of the sample glacier (Baltoro Glacier). (a) True-colour image from Landsat; (b) terrain slope of the glacier. The red line denotes the glacier boundary, which is based on the Randolph Glacier Inventory (RGI) v6.0
